# Data supporting the role of the non-glycosylated isoform of MIC26 in determining cristae morphology

**DOI:** 10.1016/j.dib.2015.04.014

**Published:** 2015-05-18

**Authors:** Sebastian Koob, Miguel Barrera, Ruchika Anand, Andreas S. Reichert

**Affiliations:** a Mitochondrial Biology, Buchmann Institute of Molecular Life Sciences, Goethe University Frankfurt, Max‐von‐Laue‐Str. 15, 60438 Frankfurt am Main, Germany; b Institute of Biochemistry and Molecular Biology I, Heinrich Heine University, Medical Faculty, Universitätsstr. 1, 40225 Düsseldorf, Germany

## Abstract

Membrane architecture is crucially important for mitochondrial function and integrity. The MICOS complex is located at crista junctions and determines cristae membrane morphology and the formation of crista junctions. Here we provide data of the *bona fide* MICOS subunit MIC26 for determining cristae morphology. MiRNA-mediated downregulation of MIC26 results in higher protein levels of MIC27 and in lower levels of Mic10. Using a miRNA-resistant form to MIC26 we show that this effect is specific to MIC26. Our data further demonstrate that depletion of MIC26 primarily affects the level of the 22 kDa mitochondrial isoform of MIC26 but not the amount of the secreted 55 kDa isoform of MIC26. Depletion of MIC27, however, increases secretion of the latter isoform. Overexpression of a myc-tagged version of MIC26 resulted in altered cristae morphology with swollen and partly vesicular cristae-structures.

## Specifications table

**Table:** 

Subject area	Biology
More specific subject area	Ultrastructure defining complexes in mitochondria
Type of data	Figures
How data was acquired	Western Blot and Electron microscopy
Data format	Analyzed
Experimental factors	Cell culture and harvesting was performed as described elsewhere [1]
Experimental features	Western-Blot signals were detected and quantified using the ChemiDoc XRS+ system and the corresponding Image Lab quantification software version 4.0.1 (BioRad).
	EM specimens were inspected with a Transmission Electron Microscope (Hitachi, H600) at 75 kV. Bioscan model 792 (Gatan) was used for image acquisition
Data source location	1) Buchmann Institute of Molecular Life Sciences, Goethe-University Frankfurt, Max-von-Laue-Street 15, 60438 Frankfurt am Main, Germany 2) Institute of Biochemistry and Molecular Biology I, Medical Faculty, Heinrich Heine University, Universitätsstr. 1, 40225 Düsseldorf, Germany
Data accessibility	Data supplied with this article

## Value of the data

• Specificity of a *MIC26* targeted miRNA is reported.
• Description of MIC26 and MIC27 as parts of a common regulatory pathway.
• Data showing the effect of *Myc-MIC26* overexpression on cristae morphology.

## 1 Data, materials and methods

MIC26 depletion induces a number of physiological impairments in mitochondria including a decrease in respiration and an altered mitochondrial ultrastructure [1]. Here we show data to demonstrate that the miRNA used in Koob et al. is specific to MIC26. For that a miRNA-resistant form of N-terminal myc-tagged MIC26 was used.

**Fig. 1:**
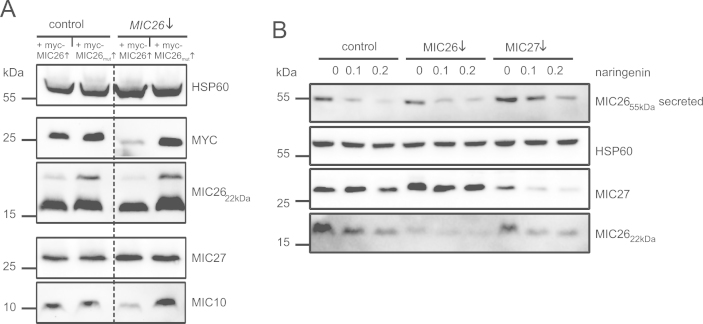
MIC26 specific effects on other MICOS subunits in MIC26-depleted cells. (A) Expression of a miRNA resistant form of myc-MIC26 (*myc-MIC26*_*mut*_) to investigate *MIC26* miRNA specificity. Western Blot performed using indicated antibodies with HSP60 as a loading control. (B) Western blot analysis of endogenous MIC26_22 kDa_ and MIC27 protein levels as well as secreted MIC26_55 kDa_ levels in miRNA expressing 143B cells. Indicated naringenin concentrations were applied to block MIC26_55 kDa_ secretion.

Fig. 1A shows that expression of the conventional *myc-MIC26* construct in MIC26↓ cells resulted in a decrease of myc-MIC26 and endogenous MIC26 levels. Likewise, protein levels of MIC27 were increased and those of MIC10 were decreased as reported before [1]. When the miRNA-resistant form was expressed in cells constitutively downregulated for MIC26 protein levels of myc-MIC26 were not reduced demonstrating that the expressed miRNA does not target these particular *myc-MIC26* transcripts. Moreover, MIC26 specific effects on MIC27 or MIC10 were not observed using the miRNA resistant myc-MIC26 construct (Fig. 1A).

We have previously demonstrated that relative levels of mitochondrial MIC26_22 kDa_ and levels of secreted MIC26_55 kDa_ can vary depending on the cell line investigated [1]. Here we show data deciphering whether depletion of MIC26 or MIC27 induces alterations in secreted levels of MIC26_55 kDa_. Fig. 1B shows MIC26 depletion in 143B cells with little influence on protein levels of secreted MIC26_55 kDa_; here, depletion of MIC26 predominatly decreases the levels of its mitochondrial isoform (Fig. 1B). In contrast, protein levels of secreted MIC26_55 kDa_ were markedly increased in 143B cells depleted for MIC27. Based on these findings we suggest that MIC27 is part of a regulatory pathway determining the amount of MIC26_22 kDa_ within mitochondria and the amount of MIC2_55kDa_ being secreted.

**Fig. 2:**
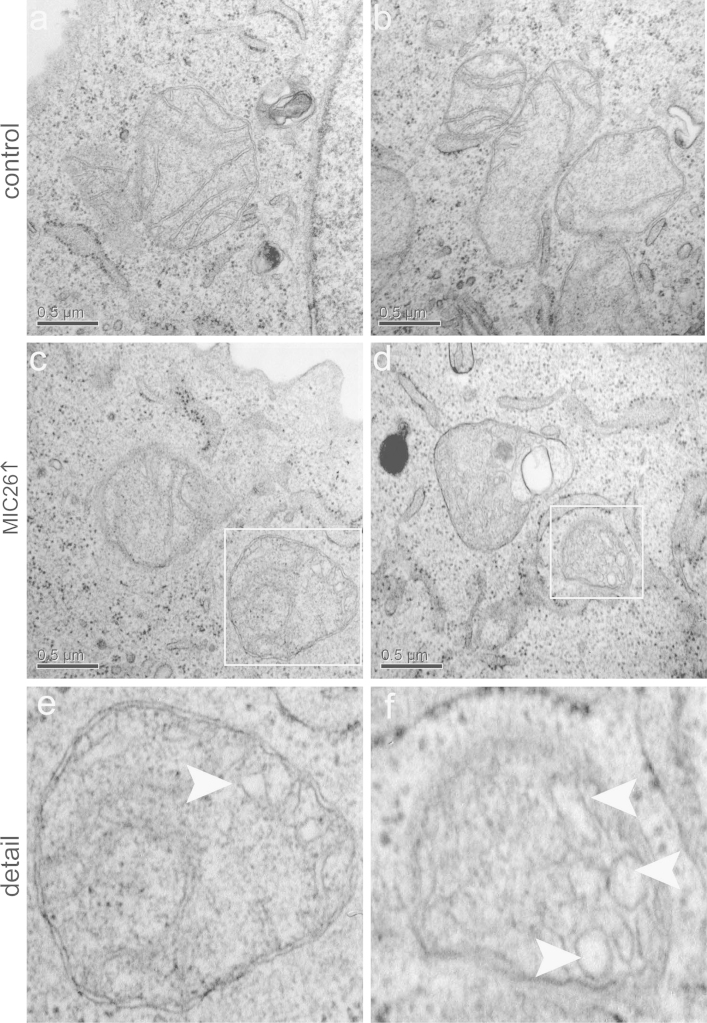
Overexpression of *myc-MIC26* leads to swollen and/or vesicle-like cristae membranes. Electron micrographs showing mitochondria from HeLa cells transfected with control plasmid (a and b) or with *myc-MIC26* (c and d). Enlarged detail pictures represent swollen cristae membranes indicated by white arrows (e and f); Scale bars 500 nm.

Fig. 2 shows that overexpression of myc-MIC26 leads to swollen or vesicle-like cristae in numerous mitochondrial sections. In control cells conventional cristae morphology with nicely tubular and ordered membranes were observed.

### 1.1 Cell culture and constructs

143B or HeLa cells have been cultured under standard conditions and were transfected like described elsewhere [1]. For overexpression we used an N-terminal myc-tagged version of *MIC26* that was described in another study of our group [1]. To generate a miRNA resistant form of *myc-MIC26* we performed site-directed mutagenesis generating silent mutations within the miRNA hybridization region of *MIC26* using the Q5 SDM Kit (NEB) according to the manufacturer´s instructions. The following primers against nucleotide positions 406–426 of the coding sequence of *MIC26* were used: forward_CCTCCGGGATTCATGGGATTAGCTGCC; reverse_ GTAAACGAGCTTCTTTATTTTTGAACCTCTAGC.

### 1.2 SDS-PAGE and western blotting

SDS-PAGE and western blot analysis was carried out with subsequent immunological detection [1] and western blot signals were detected and quantified using the ChemiDoc XRS+ system and the corresponding Image Lab quantification software version 4.0.1 (BioRad).

### 1.3 Electron microscopy (EM)

HeLa cells were transfected with a control or myc-MIC26 plasmid using *Effectene* transfection reagent (Qiagen, Hilden) and chemically fixed with 3% glutaraldehyde buffered in 0.1 M sodium cacodylate buffer, pH 7.2. Cells were harvested with a cell scraper, washed with 0.1 M sodium cacodylate buffer, pH 7.2, and embedded in 2% agarose. Staining was performed with 1% osmium tetroxide for 50 min and with 1% uranyl acetate/1% phosphotungstic acid for 1 h. Dehydration of samples was done using graded acetone series. Specimens were embedded in spurr epoxy resin and incubated for polymerization at 65 °C for 24 h. Sections were inspected with a Transmission Electron Microscope (Hitachi, H600) at 75 kV. Bioscan model 792 (Gatan) was used for image acquisition.
